# Editorial: Methods and protocols in sensory neuroscience

**DOI:** 10.3389/fnhum.2023.1291987

**Published:** 2023-09-26

**Authors:** Givago S. Souza, Thiago P. Fernandes, Natanael A. Santos

**Affiliations:** ^1^Tropical Neurology Lab, Federal University of Para, Pará, Brazil; ^2^Cognitive Neuroscience and Behaviour Programme, Federal University of Paraiba, Paraíba, Brazil

**Keywords:** sensory processing, sensory integration, neural mechanisms, assessment tools, methods

Have you ever wondered how our brains process the world around us? Understanding this is like appreciating and reflecting upon a captivating painting. Emphasizing the importance of sensory processing is essential for our daily lives. Imagine touching a hot surface; in an instant, your senses swiftly detect the danger, prompting you to reconsider and quickly move your hand away. This immediate reaction is just one illustration of how our senses gather information from the environment and transform it into signals that travel our brain's pathways. This becomes even more important for handling this complexity, much as the brush of an artist adds depth to a canvas.

The continuous advancements in sensory neuroscience not only deepen our understanding of how the brain processes sensory information at various stages but also reveal how these sensory systems interact with other parts of the body, including motor skills, autonomic responses, and other functions. Furthermore, several sensory assessment methods and protocols are now employed in monitoring human health, serving as markers to characterize or describe the functioning of the nervous system, particularly when susceptibility to conditions is a significant matter of investigation.

In our Research Topic “*Methods and Protocols in Sensory Neuroscience*,” we encouraged researchers to explore innovative approaches that could offer insights applicable to problem-solving. This compilation of five studies effectively fulfills these objectives and establishes a groundwork for investigating taste, vision, hearing, and olfaction.

Two noteworthy contributions, one by Zhao et al. and the other by Lima and Ventura, focused on visual investigation methods. Zhao et al. explored chromatic pupillometry to isolate responses from intrinsically photosensitive ganglion cells in patients with advanced retinitis pigmentosa. This non-invasive technique, increasingly utilized to identify subcortical visual pathways, presented challenges in discerning responses related to different photoreceptors. The study successfully isolated intrinsically photosensitive ganglion cell responses, highlighting their role in pupillary contraction regulation. Lima and Ventura conducted a comprehensive review of eye-tracking methods in psychophysical experiments, covering various measures and their application in stimuli detection. Their deliberations encompassed the use of eye tracking as a measure for stimulus detection, along with descriptions of four categories of measures employed as dependent variables in psychophysical experiments (i.e., reactive saccade, fixation-based measures, pursuit-based measures, and reflex-based measures).

Ninenko et al. introduced an experimental paradigm that evaluated electroencephalographic responses during tasks guided by olfactory stimuli. In this endeavor, the authors cleverly integrated visual, auditory, and olfactory cues, resulting in an environment where subjects experienced a seamless sensory fusion. The experiment comprised intervals of controlled ventilation and olfactory inhalation guided by visual cues, coupled with decision-making processes associating delivered odors with visual symbols. The authors presented a compelling array of data, affirming the potential of their proposed method as an invaluable tool for scrutinizing neural odor processing in humans.

Krasnoff and Chevalier made noteworthy contributions by reporting the application of an inaudible binaural beats protocol to induce relaxation in four stressed participants. Employing electroencephalography, they measured parameters indicative of the participants' relaxation states. The binaural beats protocol demonstrated its efficacy in modulating brain activity toward the predicted state of relaxation.

Finally, we should not disregard the insightful commentary by Ennis on the study of human sweet taste modeling. Ennis has added a valuable dimension to the discourse initiated by von Molitor et al. (2021) by elucidating how models developed in pharmacokinetics have significantly contributed to our understanding of chemosensory psychophysics.

These methods find application in both basic research and clinical studies. In the sphere of basic research, psychophysics and electrophysiology serve as indispensable tools, facilitating the elucidation and characterization of stimuli, receptive domains within the sensory cortex, and the functions of individual neurons (e.g., texture and pattern discrimination can contribute to haptic technology or product design). In clinical research, techniques such as electrophysiology and computational modeling emerge as invaluable diagnostic instruments for the assessment of sensory disorders (e.g., investigation of the ability to identify or detect stimuli in neurodegenerative disorders may help identify sensory markers or early diagnostic tools). The use of these methods and protocols corroborates established theories, refines methodological approaches, and furnishes empirical evidence for the role of sensory processing in advancing our comprehension of the complexities of the human brain.

We hope that this Research Topic encourages exploration and innovation in the field, ultimately leading to an enhanced understanding and prognosis of sensory-related conditions and disorders and more effective treatment.

## Author contributions

GS: Supervision, Validation, Visualization, Writing – original draft. TF: Supervision, Validation, Visualization, Writing – original draft. NS: Supervision, Validation, Visualization, Writing – original draft.
